# Microfluidic Characterization of Red Blood Cells Microcirculation under Oxidative Stress

**DOI:** 10.3390/cells10123552

**Published:** 2021-12-16

**Authors:** Nadezhda A. Besedina, Elisaveta A. Skverchinskaya, Alexander S. Ivanov, Konstantin P. Kotlyar, Ivan A. Morozov, Nikita A. Filatov, Igor V. Mindukshev, Anton S. Bukatin

**Affiliations:** 1 Laboratory of Renewable Energy Sources, Alferov Saint Petersburg National Research Academic University of the Russian Academy of Sciences, 194021 Saint-Petersburg, Russia; besedinadezhda@gmail.com (N.A.B.); konstantin21kt@gmail.com (K.P.K.); morivan@mail.ru (I.A.M.); nikita.filatov@inbox.ru (N.A.F.); 2 Sechenov Institute of Evolutionary Physiology and Biochemistry, Russian Academy of Sciences, 194223 Saint-Petersburg, Russia; lisarafail@mail.ru (E.A.S.); iv_mindukshev@mail.ru (I.V.M.); 3 Institute of Physics and Mechanics, Peter the Great Saint-Petersburg Polytechnic University, 195251 Saint-Petersburg, Russia; a.s.ivanov@mail.ru; 4 Institute for Analytical Instrumentation of the RAS, 190103 Saint-Petersburg, Russia

**Keywords:** microfluidics, oxidative stress, red blood cells, microcirculation, biophysical phenotyping

## Abstract

Microcirculation is one of the basic functional processes where the main gas exchange between red blood cells (RBCs) and surrounding tissues occurs. It is greatly influenced by the shape and deformability of RBCs, which can be affected by oxidative stress induced by different drugs and diseases leading to anemia. Here we investigated how in vitro microfluidic characterization of RBCs transit velocity in microcapillaries can indicate cells damage and its correlation with clinical hematological analysis. For this purpose, we compared an SU-8 mold with an Si-etched mold for fabrication of PDMS microfluidic devices and quantitatively figured out that oxidative stress induced by *tert*-Butyl hydroperoxide splits all RBCs into two subpopulations of normal and slow cells according to their transit velocity. Obtained results agree with the hematological analysis showing that such changes in RBCs velocities are due to violations of shape, volume, and increased heterogeneity of the cells. These data show that characterization of RBCs transport in microfluidic devices can directly reveal violations of microcirculation caused by oxidative stress. Therefore, it can be used for characterization of the ability of RBCs to move in microcapillaries, estimating possible side effects of cancer chemotherapy, and predicting the risk of anemia.

## 1. Introduction

One of the main current trends in the development of microfluidic devices is “organs-on-a-chip” [1,2,3]. Such devices make it possible to simulate the processes occurring in natural conditions in the body of animals and humans. This makes it possible to study the effect of drugs on physiological processes in the body, test their effectiveness and toxicity, and select the optimal doses of drugs for a particular patient, all while minimizing side effects [4]. At present, lung-on-a-chip [5,6,7], kidney-on-a-chip [8,9,10], and brain-on-a-chip devices [11] have already been demonstrated to simulate these organs effectively.

One of the main functional systems of the body that ensures its functioning is the circulatory system. Its main part is erythrocytes (or red blood cells, RBCs), cells responsible for gas exchange throughout the body. One of the key functional features of RBCs is their ability to adapt their shape to dynamic flow conditions. It leads to their ability to move in microcapillaries, in which the main gas exchange between cells and surrounding tissues takes place [12,13]. RBC deformability results from several cytological parameters such as membrane mechanical properties, cytoplasmic viscosity, and surface area to volume (S/V) ratio [12]. Changes in the characteristics of any of these parameters, be it an increase in vesiculation, a change in the operation of ion channels and pumps, or a change in hemoglobin concentration will lead to a change in the deformability of RBCs [14]. It, in turn, will lead to a deterioration of gas exchange in tissues and organs and the occurrence of anemia [15]. Since anemia is a consequence of many diseases and is also one of the main side effects of chemotherapy for oncological diseases, the development of express methods for analyzing the deformability of RBCs and simulation of microcirculation in vitro is an important and urgent task. The solution will minimize the risks of anemia and increase the effectiveness of personal treatment [14].

Currently, several methods that directly assess the deformability of cells have been developed, including aspiration into a micropipette, optical stretching, and atomic force microscopy [16,17,18,19]. However, these methods have low productivity and high labor intensity, which does not allow their use in clinical practice. Microfluidic devices make it possible to directly simulate blood microcirculation in vitro with a high rate of statistics collection [20]. At the moment, three methods of mechanical phenotyping of cells in microfluidic devices have been developed: constriction-based deformability cytometry [21,22,23,24], shear flow deformability cytometry [25], and extensional flow deformability cytometry [26]. The first method records the time taken by the cell to pass the constriction in the microfluidic chip. The other two methods register the change in the shape of the cells under the influence of the shear rate of the flow.

Microfluidic analysis of the RBCs deformability from different donors during blood storage allows optimizing its storage time [27] since a violation of the deformability of bank erythrocytes can affect blood microcirculation [28]. Disturbance of deformability of RBCs infected with malaria plasmodium underlies the hydrodynamic separation of cells, which contributes to an earlier diagnosis of this disease [29]. Recently a microfluidic assay to measure RBCs occlusions under various conditions in vitro was developed to evaluate the clinical efficiency of different drug therapy [30]. Moreover, microfluidic analysis has been successfully used for the biophysical characterization of sickle cell erythrocytes, allowing in vitro modeling of an oxygen gradient, a trigger factor for developing occlusive crises during polymerization of hemoglobin S in these patients [31].

In this work, we investigate the effect of the geometric dimensions of the microfluidic microchannels and the roughness of their walls on the RBCs velocity under different oxidative stress conditions caused by exposure to *tert*-Butyl hydroperoxide (tBuOOH). To vary these parameters of the microfluidic devices, we compare the capabilities of the two soft lithography mold fabrication technologies, namely SU-8micropatterning and direct silicon etching. To explain the changes in the cell’s velocities, we performed their hematological analysis, which showed an increase in cell volume and spherization. These changes led to the appearance of a subpopulation of cells with significantly low velocity in the microchannels of the device. The data obtained will allow the development of more efficient microfluidic devices for simulating microcirculation to analyze the effect of oxidative stress on red blood cells and improve personal strategies of cancer chemotherapy.

## 2. Materials and Methods

### 2.1. Sample Collection and Preparation

Blood samples were obtained from 14 healthy donors (8 women and 6 men, ages 24–67) by puncturing the ulnar vein in vacuum monovettes-s with a 3.2% sodium citrate anticoagulant (Sarstedt, Nümbrecht, Germany). Erythrocytes were separated from plasma by centrifugation at 400× *g* 3 min (Centrifuge ELMI-50CM, Elmi, SIA Elmi, Latvia), and washed twice in HEPES-buffer, mM: NaCl—140, KCl—5, MgCl2—3, glucose—5, HEPES—10, EGTA—2, pH 7.38 (pH-meter Mettler Toledo), 295–300 mOsm/kg H_2_O (Osmomat 030 cryoscopic osmometer, Gonotec, Berlin, Germany), under the same centrifugation regime.

The concentration of erythrocytes in whole blood and cell suspension in buffer (red blood cells, RBC), mean cells volume (MCV, fL), and population heterogeneity by volume (RDW%) were controlled on a Medonic-M20 hematology analyzer (Boule Medical A.B., Stockholm, Sweden).

Simulation of oxidative stress (OS) conditions was performed by incubation of (RBCs suspension 0.5 × 10^9^ cell/mL with a widely used and well characterized oxidizing agent organic peroxide *tert*-Butyl hydroperoxide (tBuOOH, Sigma-Aldrich, Munich, Germany) in a Biosan-100 thermoshaker (BioSan, Latvia) for 4–5 h at 37 °C shaking at 420 rpm [32,33,34,35,36]. tBuOOH is an amphiphilic oxidizer, which causes complex damage both in the lipid layer of the membrane and in the cytosol of the cell. Additionally, it is an initiator of free radicals (peroxide and alkoxyl radicals), which cause the formation of reactive oxygen species (ROS) that induce futher cell damage.

### 2.2. Fabrication of Microfluidic Devices

Microfluidic devices were made from polydimethylsiloxane (PDMS) by standard soft lithography technique [37,38] using two different types of silicon molds. The first one was fabricated by contact photolithography with a chromium mask in a SU-8 2005 layer on a silicon wafer. The second one was fabricated by cryogenic reactive ion etching of silicon wafer through a chromium mask formed on the wafer by lift-off lithography. The depth of microchannels on both molds was 8 µm. Their dimensions were measured by scanning electron microscope Supra 25 (Carl Zeiss, Oberkochen, Germany) and by a high-resolution stylus-type profilometer XP-1 (Ambios Technology, Santa Cruz, CA, USA). Before the measurements, the mold with SU-8structures was covered by 5 nm chromium film using e-gun sputtering.

PDMS replicas were obtained by curing a degassed mixture of Sylgard 184 Silicone Elastomer Base and the Curing Agent 10:1 (Dow Corning, Midland, TX, USA) poured onto both molds and cured at 65 °C, 4 h in an oven. After separating the PDMS replica from the mold, inlet and outlet holes were cut out using a 1 mm biopsy puncher. Then they were treated by oxygen plasma and covalently bonded to glass slides. Tygon tubing was used to introduce cells into the devices. The injection of RBCs (5 × 10^7^ cells/mL) into the microchip was performed at constant pressure, which allowed maintaining an equal rate of cells in all microchannels even when part of them was occlused.

### 2.3. Microfluidic Experimental Procedure and Image Analysis

Data for the analysis of erythrocyte transport in the microchannels were obtained by recording videos at 400–450 fps with a XIMEA MC023MG-SY camera (XIMEA GmbH, Münster, Germany) using a Leica DM4000B LED microscope (Leica Microsystems GmbH, Wetzlar, Germany) with 20×/0.40 objective lens (Leica Microsystems, Wetzlar, Germany). A region of interest containing one microchannel was selected to make the recording. During every experiment for each concentration of tBuOOH, 9–10 channels were recorded. The number of cells analyzed in each type of microchannels at each concentration of tBuOOH is presented in Table 1.

A custom script developed in the MATLAB software package (The MathWorks) was used to calculate the velocity of RBCs in the microchannels. It determined the frames in which the cell entered and exited a channel. The difference in frames was used to calculate the velocity of the cell passage. This velocity was normalized to the velocity of the fluid flow in the channel measured by analyzing cells velocities in a wide channel before and the measurement microchannel. The data of each experiment were processed in OriginPro 2021b (OriginLab Corporation). First, the data were converted to the form of probability density (Statistics—Frequency Counts), and then they were averaged and approximated by the Gaussian function.

### 2.4. Osmotic Fragility Test

Changes in the shape of RBCs under oxidative stress were assessed by low angle light scattering on a LaSca-TM microparticle analyzer (BioMedSystems LLC, St. Petersburg, Russia) [39]. The incubated erythrocytes were resuspended in HEPES buffer and transferred to the analyzer’s cuvette. The RBCs concentration was 1.0 × 10^6^ cells/mL, which provided registration of light scattering from single cells [40]. The LaSca method is based on measuring the light scattering profile of a sample. The intensity profile of scattered light depends on the scatter angle and correlates with the size and shape of the observed particles. By providing conditions of single scattering according to the Mie description of the scatter process, the LaSca technique allows for simultaneous measurement of changes of particle size and shape. To maintain single scattering conditions, the cells’ density must be adjusted to yield a transmittance of at least 50% of the transmittance of the cuvette alone. Recording the intensity of light scattering was performed at a physiological value of the buffer osmolality 300 mOsm. During the test, we obtained the discoid ratio (the proportion of spherized erythrocytes), calculated from the amplitude of osmoscan oscillations.

### 2.5. Statistics

The Student’s *t*-test, 2-taled, was used to compare data between groups. When comparing data between microchips of different sizes, an unpaired t-test was used. The paired test was used to compare the data for tBuOOH exposure. Significant differences were recorded at *p* ≤ 0.05. The following programs were used for data processing: video file processing—MATLAB (The MathWorks); laser diffraction data—the built-in program of the LaSca-TM analyzer (Biomedical systems, Russia); statistical processing—Excel 2016 (Microsoft Corporation), OriginPro 2021b (OriginLab Corporation) and GraphPad Prism 8 (San Diego, CA, USA).

## 3. Results and Discussion

### 3.1. Design of Microfluidic Chip

**Figure 1:**
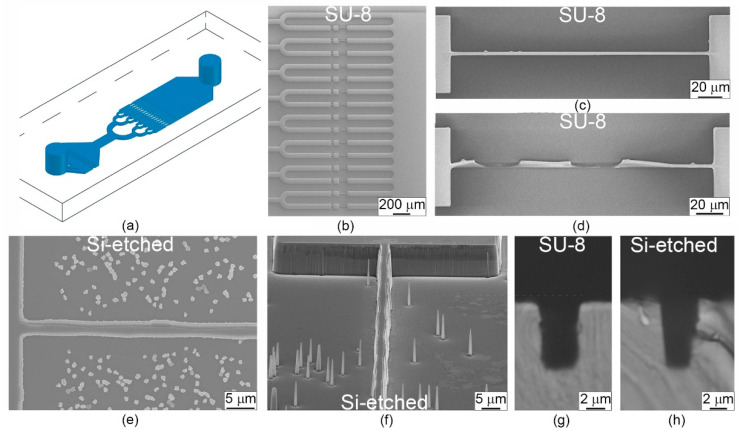
Microfluidic device for mimicking microcapillaries of the circulation system: (**a**) schematic view; (**b**) total view of 16 microchannels on the SU-8 mold; (**c**) example of a SU-8 microchannel on the new mold; (**d**) example of a SU-8 microchannel after three months of usage; (**e**) top view and (**f**) side view of a channel on the Si-etched mold; (**g**) cross section of a PDMS replica fabricated with SU-8 mold; (**h**) cross section of a PDMS replica fabricated with Si-etched mold. All images were obtained by scanning electron microscope.

To study the RBCs microcirculation, we developed a microfluidic device containing 16 parallel microchannels with a depth of 8 μm and an average width of 1.5–3 μm (see Figure 1a,b). To reliably determine the transit time of the cells, we chose the length of all channels of 200 µm. To reduce the distribution of the cell’s transit time in the microchannels, we added the aligning channels in front of the measurement channels, which were 5 µm wide and 30 µm long [41]. To fabricate these devices from PDMS using soft lithography, we used silicon molds, in which the relief was formed in a SU-8 photoresist or was made in the silicon itself by reactive ion etching. Both of these methods can provide the aspect ratios of microstructures up to 10:1.

Within this work, the first mold was made by photolithography in an SU-8 resist and the second one by reactive ion etching of silicon. Microchannels 1.5, 2, and 3 µm wide were formed in the SU-8 resist (SU-8 mold) (see Figure 1c,d), while in silicon (Si-etched mold), the average channels width was 2.5 and 3 µm (see Figure 1e,f). The side walls of the microstructures in SU-8 were close to vertical (the angle is 90°). Therefore, the channels cross section was rectangular, and their width differed from the specified values by less than 0.1 μm (see Figure 1g). When the mold was made by direct silicon etching, the inclination angle of the microstructures’ side walls was ~86°, which led to a trapezoidal cross section and an increase in the channel’s width at half-height by 1 μm as compared to the initial design (see Figure 1h). Additionally, the non-verticality of side walls led to the creation of needle-like nanostructures on the substrate, which, however, did not affect the cells transport in the microchannels. Besides, according to the electron microscopy images, it can be seen that the vertical walls of the microstructures in SU-8 have a lower roughness compared to Si-etched ones.

Both molds were used to fabricate several dozens of PDMS microfluidic devices for experimental studies. After that, they were inspected for changes щa the size of the microchannels. On the silicon mold, the channels did not change their size and shape, while more than 50% of the microchannels 1.5 and 2 µm wide on the SU-8 mold were damaged (see Figure 1d). It happened since SU-8 is an epoxy-based photoresist, which elasticity is much lower than the elasticity of silicon [42]. In addition, the adhesion of the SU-8 layer to the silicon wafer might have an important influence on the destruction process. The heating/cooling of the mold during the PDMS curing process and further replica detachment increased the deformation and damage of the microstructures on the mold.

### 3.2. Study of the RBCs Transport in Microchannels under Oxidative Stress

**Figure 2:**
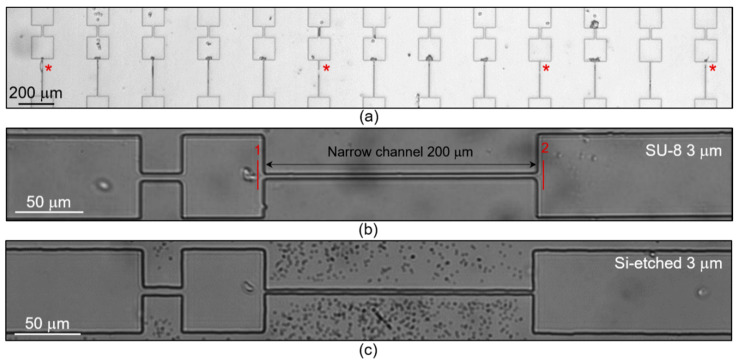
RBCs transport in microfluidic channels: (**a**) total view of occluded 1.5 µm wide channels by RBCs, treated with 0.5 mM of tBuOOH (* mark the damaged channels); (**b**) example of a 3 µm wide microchannel made by SU-8 mold (the marks indicate recognition points for the script to determine RBCs velocities); (**c**) example of a 3 µm wide microchannel made by Si-etched mold. All images were obtained by a brightfield optical microscope.

**Figure 3:**
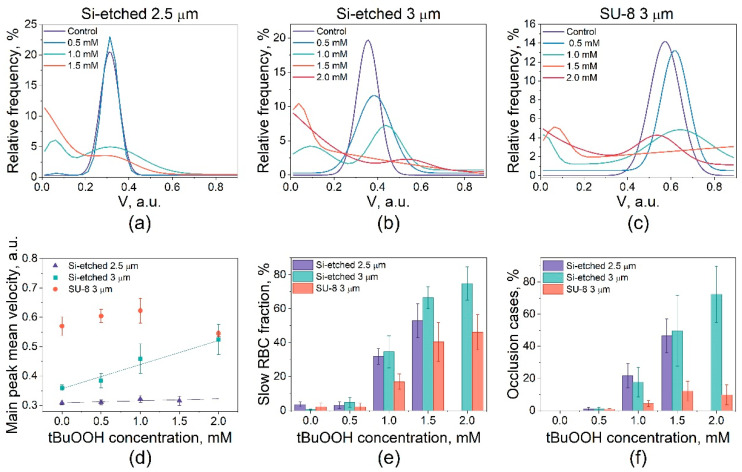
Velocities of RBCs exposed to oxidative stress in different microfluidic channels: (**a**) rough 2.5 μm wide microchannels made with the Si-etched mold; (**b**) rough 3 μm wide microchannels made with the Si-etched mold; (**c**) smooth 3 μm wide microchannels made with the SU-8 mold; (**d**) the main peak mean velocity according to the tBuOOH concentration; (**e**) slow RBCs fraction ratio according to tBuOOH concentration. (**f**) Percentage of occlusions cases in microchannels of different widths and roughness. All data are presented as mean ± SE.

The velocity of RBCs in microchannels depends on both the size of the cells and the size of the microcapillaries. Human RBCs have the form of biconcave discs with a diameter of 6.2–8.2 µm and a maximum thickness of 2–2.5 µm [43]. It was found that erythrocytes were unable to efficiently move in microchannels 1.5 μm wide due to massive occlusions (*n* = 3 donors, see Figure 2a). In wider microchannels, native erythrocytes can move efficiently with a probability of occlusions close to zero (see Figure 2b,c). In this case, the distribution of the RBCs relative velocities can be described by a Gaussian function with an average velocity of 0.31–0.58 a.u., depending on the width and roughness of the channels. The absolute values of these velocities were 6–10 mm/s, which is comparable with in vivo studies [44]. Due to the smoother side walls, the cells’ relative velocities in the channels made with the SU-8 mold were significantly higher (see Figure 3).

To select the optimal microchannels for simulation of blood microcirculation, we studied the RBCs velocities under the action of oxidative stress caused by tBuOOH at concentrations 0.5–2.0 mM. At tBuOOH concentration of 0.5 mM, the distribution of cell velocity in microchannels continued to be described by the Gaussian function (see Figure 3a–c). With an increase in the oxidant concentration, the distribution of velocities changed significantly and became bimodal. The main mode corresponded to normal cells, the velocity of which changed insignificantly compared to the control. The second mode responded to damaged slow cells, the velocity of which was notably lower than that of the control. With an increase in the concentration of tBuOOH, the number of normal cells decreased, and the number of slow cells increased. Thus, the population of slow cells corresponded to cells damaged by oxidative stress. Such behavior of the erythrocyte velocity distribution function was typical for all the investigated microchannels. In both types of microchannels of 3 μm wide made by SU-8 and Si-etched mold, the average velocity of normal cells changed with an increase in the tBuOOH concentration, while in Si-etch microchannels 2.5 μm wide with rough walls, it did not change (see Figure 3d). Also, in 3 μm wide channels with smooth walls (SU-8 mold), the number of slow cells (see Figure 3e) and the probability of occlusions (see Figure 3f) were less than in channels with rough walls (Si-etched mold). Such behavior of the cells is due to the fact that the friction force of cells against the walls depends on their roughness, which affects their speed and the occlusions probability.

### 3.3. Hematological Analysis

**Figure 4:**
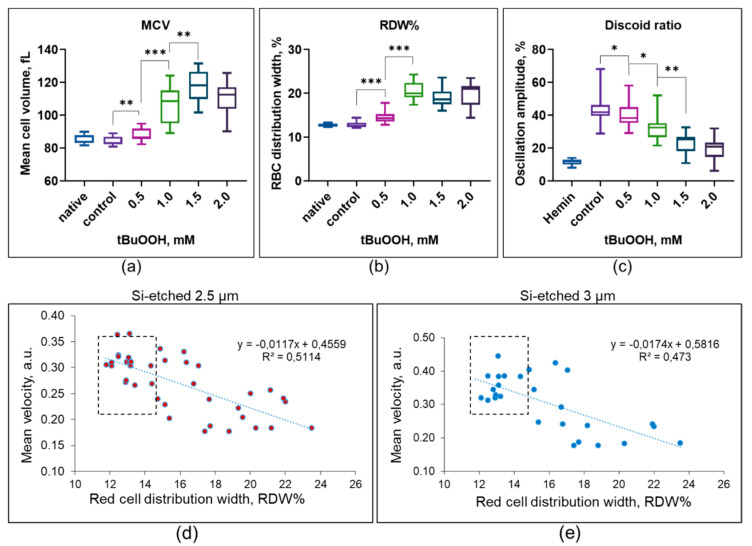
Hematological analysis of erythrocytes exposed to oxidative stress caused by tBuOOH show: (**a**) increase of mean cell volume; (**b**) increase heterogeneity of the population by volume; (**c**) decrease of low angle light scattering oscillations indicating increase in RBCs spherization. * *p* < 0.05; ** *p* < 0.01; *** *p* < 0.00; 2-tailed independent samples, *t*-test, *n* = 14 donors. (**d**) The relationship between the RBCs mean velocity in microchannels and the indicator of the heterogeneity of the RBC population by volume, RDW% in 2.5 µm-wide channels and (**e**) in 3 µm-wide channels; RDW%—hematological analyzer data; the dashed field shows the values for RDW% and mean velocity of untreated by tBuOOH cells.

Hematologic analysis was performed to determine the structural changes in RBCs caused by oxidative stress that led to the appearance of slow cells. The obtained data showed that exposure of RBCs to tBuOOH led to an increase in the mean volume of RBCs (MCV). Even at tBuOOH concentration of 0.5 mM, the increase in MCV was significant (84.2 ± 2.7 fL vs. 88.5 ± 4.3 fL, *p* < 0.02, *n* = 14 donors), although its absolute values did not go beyond the normal range. At the concentration of tBuOOH of 1.5 mM, MCV was 1.48 times higher than the control values (118.6–130.3 fL, *n* = 14 donors) and significantly exceeded the range of healthy person’s values (80–96 fL) (see Figure 4a). In addition, oxidative stress changed the distribution width of the RBCs volume, which is described by the MCV distribution width at half maximum, expressed by the parameter RDW%. For healthy people, the RDW% is 11.0–14.5%. The increase in RDW% at the concentration of tBuOOH of 0.5 mM was 14.7 ± 1.73 against the control level of 12.7 ± 0.35, *p* < 0.000 (*n* = 14 donors, see Figure 4b).

Analysis of the RBCs shape was carried out by the low angle light scattering technique using the analyzer LaSca-TM [39]. Since the intensity of laser radiation scattering by cells is an indirect indicator of cell shape, we used fully spherized cells obtained by treating them with hemin as a positive control [45]. The analysis showed that under the action of oxidative stress, RBCs lost their discoid forms, which led to an increase in the proportion of spherized cells (*n* = 14 donors, see Figure 4c).

The obtained results show that under oxidative stress, RBCs may lose control over the regulation of their volume and shape, which leads to a loss of the ability to move in microchannels. It affects the cells unevenly, which can be explained by the initial heterogeneity of the RBCs biophysical parameters due to the presence in the circulation of cells of different ages and their accumulated damage caused by limited reparation possibilities [46,47]. According to this, our results fully fit into the modern concept of the multiplicity of responses of the population of erythrocytes to external influences.

In addition, an increase in the width of the distribution of cell volume RDW% indicates that different cells in the population have different resistance to oxidative stress. An increase in RDW% has been observed in various diseases and is now considered as an important predictive tool for the patient [48,49,50]. Our experiments show that the RDW% strongly correlates with the cell’s mean velocities in microchannels (Figure 4d,e), which means that it can be used for indirect estimation of the ability of RBCs to move in microcapillaries.

## 4. Conclusions

RBCs’ biophysical phenotyping is of great practical importance [51,52]. It has already been shown that microfluidic analysis can successfully register RBCs disorders [21,31,41,53]. For improving the analysis of the functional characteristics of RBCs, the microfluidic device must provide high sensitivity to deviations from the physiological norm and establish a standard experimental procedure. Therefore, it is necessary to determine limitations associated with the technical characteristics of the microfluidic devices.

In our work, we focused on the characterization of the behavior of RBCs in microchannels and how their dimensions, roughness, and cross section influence RBCs transit velocity under oxidative stress induced by tBuOOH. For this purpose, we used two types of silicon molds for the fabrication of microfluidic devices with microchannels, which dimensions are comparable with the size of the cells. The SU-8 mold provided rectangular cross section and smooth side walls of the channels, while the silicon etched mold provided trapezoidal cross section and rough side walls. Unlike the Si-etched mold, the SU-8 mold is less strong and can be damaged during the PDMS replica detachment.

Our results show that RBCs are not able to successfully move in PDMS 1.5 μm wide microchannels due to massive occlusions. In all of the other channels, the occlusions probability is close to zero, and the cells’ relative transit velocity can be approximated by the Gaussian function. Its mean velocity is higher in those channels, which are wider and have smooth side walls. Under oxidative stress induced by tBuOOH, the whole population of RBCs splits into the subpopulations of normal and slow cells. Hematological analysis showed that such changes in RBCs transport are due to violations of shape, volume, and increased heterogeneity of the cells. The transit velocity of normal cells is close to the velocity of untreated ones in control, while the transit velocity of slow cells is significantly lower. This indicates that cells damaged by oxidative stress lose the ability to effectively pass narrow microchannels and increase the probability of their occlusion. These trends were the same in all channels. However, the absolute values of velocities and occlusion cases strongly depended on the channel’s width and side walls roughness. Therefore, microfluidic devices with different channels dimensions require calibration to provide quantitative results.

Obtained results show that characterization of RBCs transport in microfluidic devices can directly reveal violations of microcirculation caused by oxidative stress. Therefore, this method can be used for simulating microcirculation under various conditions, estimating possible side effects of cancer chemotherapy, and predicting the risk of anemia.

## Figures and Tables

**Table 1:** The number of analyzed cells in each experiment.

Channels Size	Number of Donors	Control	tBuOOH				Total
			0.5 mM	1 mM	1.5 mM	2.0 mM	
1.5 µm (SU-8)	3	516	427	-	-	-	943
2.5 µm (Si-etched)	5	10,834	8450	4438	3194	184	27,100
3 µm (Si-etched)	3	5188	4886	3915	1446	674	16,109
3 µm (SU-8)	3	1451	1771	1575	736	294	5827

## Data Availability

The data presented in this study are available on request from the corresponding author.
